# Low Incidence of hepatic sinusoidal obstruction syndrome/veno-occlusive disease in adults undergoing allogenic stem cell transplantation with prophylactic ursodiol and low-dose heparin

**DOI:** 10.1038/s41409-021-01546-w

**Published:** 2022-01-04

**Authors:** Lina Stutz, Jörg P. Halter, Dominik Heim, Jakob R. Passweg, Michael Medinger

**Affiliations:** 1 Division of Hematology, University Hospital Basel,, Basel, Switzerland; 2 University of Basel, Basel, Switzerland

**Keywords:** Risk factors, Translational research, Acute myeloid leukaemia

## Abstract

Hepatic sinusoidal obstruction syndrome (SOS)/veno-occlusive disease (VOD) is a complication after allogenic hematopoietic stem-cell transplantation (allo-HSCT) with high mortality. The purpose of this study was to assess the incidence and outcome of SOS in patients after allo-HSCT with the impact of ursodeoxycholic acid (UDCA) and low-dose heparin as SOS prophylaxis. Out of 1016 patients, 23 developed SOS, with a cumulative incidence of 2.3% (95% CI 1.3–3.3) 6 months after HSCT. Approximately one quarter of these patients (26.1%) had late-onset SOS. A high proportion were very severe SOS cases (74%), and 83% of the patients were treated with defibrotide (DF). In multivariate analysis, advanced disease (*p* = 0.003), previous HSCT (*p* = 0.025) and graft versus host disease (GvHD) prophylaxis by post-transplant cyclophosphamide (PTCy) (*p* = 0.055) were associated with the development of SOS. The 1-year overall survival (OS) was significantly lower in the SOS group compared to patients without SOS (13% versus 70%, *p* = 0.0001). In conclusion, we found a low incidence of SOS in patients receiving low-dose heparin and UDCA prophylactically, but among SOS patients, a high mortality. Low-dose heparin and UDCA might be a prophylactic approach for SOS.

## Introduction

Hepatic sinusoidal obstruction syndrome (SOS) (formerly veno-occlusive disease (VOD)) is a potentially life-threatening complication after allogenic hematopoietic stem cell transplantation (allo-HSCT) [1]. An injury of the sinusoidal endothelium of the liver seems to be the underlying cause [1, 2], triggered by several factors including conditioning, drugs, allo-immunological reactions [3], and occurrence is favored by pre-existing liver damage [4]. Due to loss of integrity of the endothelium, erythrocytes, leukocytes and cellular debris penetrate into space of Disse [3], leading to an embolization of the centrilobular veins [2], resulting in hepatocellular damage and portal hypertension [5]. Clinical manifestations are unspecific [1], with hyperbilirubinemia, weight gain, painful hepatomegaly and ascites [5, 6]. The onset can be either gradually or disruptive. The clinical course is ranging from mild forms spontaneously resolving, to severe forms with organ damage and multiorgan failure (MOF) [2]. The diagnosis is based on clinical presentation, supported by ultrasound, or, if the risk of invasive procedure is acceptable, confirmed histologically [1].

Risk factors to develop SOS may be related to the patient’s condition before transplantation (e.g. Karnofsky performance scale (KPS) less than 90%) [7, 8], to the transplant procedure (e.g. (high dose) busulfan containing regimen) [1, 9] or to the health status of the patient’s liver (e.g. high transaminases) [4].

Varying incidence of SOS has been reported in literature. An older, but one of the only available prospective studies about SOS, indicated an SOS incidence of SOS of 8.9% after allo-HSCT (children and adults) [7]. In a systemic review analyzing 135 studies on SOS (including children and adults, after allo- or auto-HSCT), a mean incidence of 13.7% was reported [10]. A more recent retrospective study in adults and children receiving their first allo- or auto-HSCT in the clinical database of the Center for International Blood and Marrow Transplant Research (CIBMTR) found an incidence of 3.2% (*n* = 275/8,341) [11].

Defibrotide (DF), a polydisperse oligonucleotide with antithrombotic, anti-ischemic and anti-inflammatory activity on microvasculature, is the only approved agent for the treatment of SOS [5]. Whereas a randomized controlled trial (RCT) investigating the efficacy of the agent is lacking, a prospective study comparing patients with SOS and MOF treated with DF (*n* = 102) with historical controls (*n* = 32) found a significantly higher day+100 survival rate (38.2% versus 25.5%), and a significantly higher SOS resolution rate on day+100 after HSCT (25.5% versus 12.5%) in patients treated with DF [12]. Strouse et al. [11] compared retrospectively two groups of patients with severe or very severe SOS, the one treated with DF (*n* = 41), the other without (*n* = 55), and found a trend towards better survival on day + 100 with DF treatment, although not significantly [11]. In an expanded-access treatment protocol, early initiation of DF has been associated with improved day+100 survival [6], indicating that prompt initiation is paramount in SOS treatment. A systematic review of DF studies yielded a day + 100 survival of 41% for patients with SOS and MOF treated with DF [13]. In a post-marketing study collecting retrospective and prospective data on patients receiving DF, day + 100 survival among very severe SOS was reported to be 43% [14]. These outcomes seem to be better than in an older study reporting a day+100 survival rate of 33% for patients with severe SOS without DF treatment [7]. DF treatment is recommended by several expert groups [5, 15, 16]. Contraindications of DF are clinically significant acute bleeding requiring blood transfusion, allergic reactions and concomitant use of thrombolytic therapy. The most frequent adverse reactions observed during DF application are hemorrhage and hypotension [17].

The role of DF as prophylaxis of SOS is not yet fully evaluated. In a pediatric randomized controlled open-label trial, the prophylactic use of DF was associated with a lower incidence of SOS by day + 30 after HSCT (allogenic and autologous), compared to the control arm (12% vs. 20% of; risk difference –7.7% [95% CI –15.3 to –0,1]; *p* = 0. 0488; log-rank test *p* = 0.0507) [18]. In the same study, patients who received DF prophylaxis had also a lower incidence of acute graft versus host disease (GvHD) by day+30 and day+100 after HSCT than the control group [18]. Results of the first prospective RCT comparing DF versus best supportive care alone in the prevention of SOS in adults undergoing HSCT are not available yet [19]. Expert groups suggest that DF prophylaxis can be considered for adult patients at very high risk of SOS [5], respectively is recommended in children at high risk for SOS [15, 16]. The high costs of DF are controversial [20]. A recent study evaluated the cost-effectiveness of DF compared to best supportive care (BSC) for the treatment of SOS with MOF after HSCT [21]. They found a lower SOS-related hospitalization duration (7.5 vs. 23.2 days) and proportional less stay in intensive care unit (30% vs 60%) in DF treated patients compared to BSC patients, which implies a better cost effectiveness in patients receiving DF. The analysis based on data of the study of Richardson et al. [12], which compared 102 patients treated with DF (between 2006 and 2008) with a historical control group (*n* = 32, treated between 1995 and 2007) and the results were adapted to the Spanish health system (2019) [21]. In an editorial about this study, Gratwohl criticizes that the study is based on data which was partly collected over 20 years ago and adapted to today’s health system. Also, he questions the high price of DF, which was inexpensive when the drug was approved in the 1980ies for prophylaxis of thrombotic complications, and only since being granted orphan drug status by the European medicine agency (EMA), in 2004, became this expensive [20].

Anticoagulation with low-dose heparin or low-molecular-weight heparin (LMWH) has previously been used for SOS prophylaxis. It is currently not recommended by expert groups given the absence of conclusive results and potential side effects [5, 15]. One meta-analysis, published in 2006, showed that prophylactic anticoagulation is associated with a non-significant decrease in risk of SOS (pooled RR 0.90 [95% CI 0.62–1.29]) [22]. The authors analyzed nine cohort studies and three RCTs, however, they point out a significant heterogeneity between the analyzed studies (e.g. auto- and allo-HSCT, time of SOS prophylaxis initiation, administration only to high risk patients, outcome definition) [22]. The risk of major bleeding was low with low-dose heparin and LMWH as SOS prophylaxis in these studies. Seven out of the 12 studies reported bleeding as an adverse event, none of these events were found to be more frequent in the anticoagulation group than in the control group [22]. In one of the three available randomized studies, the relative risk to develop SOS was significantly lower (0.18 [95% CI 0.04–0.78]) when using low-dose heparin as SOS prophylaxis, compared to no prophylaxis [23].

In contrast, the prophylactic administration of UDCA in patients undergoing HSCT is recommended [5]. In a systematic review of controlled clinical trials, the prophylactic use of UDCA was associated with a significantly reduced proportion of patients with SOS [24]. A prospective clinical trial showed as well a benefit: the administration of UDCA was associated with a significant reduction of patients with elevated bilirubin levels and severe acute GvHD, leading to a significantly lower NRM and better OS [25].

There is no literature available about a synergistic effect of UDCA and low-dose heparin. However, nineteen years ago, Park et al compared patients receiving heparin and UDCA (*n* = 82) with patients receiving heparin only (*n* = 83) as SOS prophylaxis in a prospective RCT [26]. They found no significant difference in SOS incidence: 16 (19.3%) patients were diagnosed with SOS in the heparin only and 13 (15.9%) in the heparin plus ursodiol group (*p* = 0.348). In the heparin only subgroup there were more severe SOS cases than in the subgroup with ursodiol and heparin (5 vs 2), although the difference was not statistically significant (*p* = 0.321) [26].

Heparin may interact in the pathomechanism of SOS development by reducing the procoagulant effect of injured endothelial cells, leading to less SOS occurrence and less severe SOS cases. This may have a synergistic effect with UDCA, which reduces hepatotoxic hydrophobic bile acids, leading to a liver protective effect [25].

The purpose of the present study was firstly to assess the incidence of SOS 6 months after HSCT in a retrospective cohort study from 2006 to 2020 of patients receiving UDCA and low-dose heparin as SOS prophylaxis. Secondly, to compare patients with and without SOS, with regard to patient, disease and transplant characteristics, and the clinical course (OS, PFS, NRM and relapse). Further, the incidence of SOS depending on the provision of DF as treatment or prophylaxis was examined.

## Patients and methods

### Patient population and study design

**Fig. 1:**
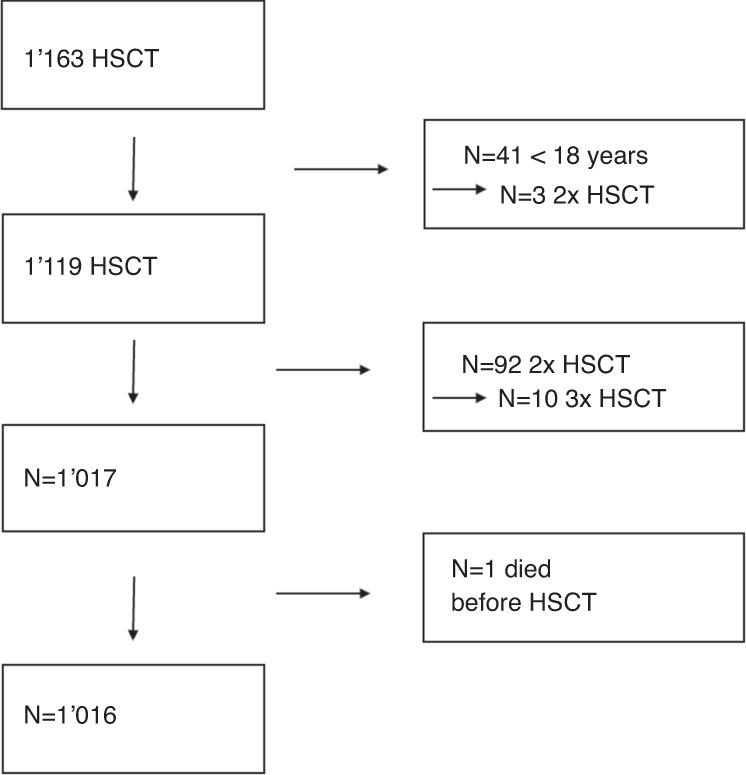
Flow diagram of enrolled patients. Inclusion procedure of patients receiving allo-HSCT at University Hospital Basel from 2006 to 2020.

This is a retrospective, single-center cohort study based on data of electronic medical records. Patients with different hematological diseases who underwent allo-HSCT between 12.01.2006 and 22.09.2020 at the University Hospital of Basel were included. Patients were excluded who were less than 18 years old (*n* = 41) or died before transplantation (*n* = 1). If patients received more than one allo-HSCT in the period of observation (*n* = 92), the last one was counted. In total, *n* = 1,016 patients were included (Fig. 1). Patient, disease and transplant characteristics were collected as potential risk factors for SOS, including age, sex, date of allo-HSCT, diagnosis, disease status before allo-HSCT, number and type of previous HSCT, performance status (KPS, Eastern Cooperative Oncology Group performance scale (ECOG)) [27], donor sex and donor type (unrelated, matched relative, haploidentical), intensity of conditioning regimen (myeloablative conditioning (MAC) or reduced-intensity conditioning (RIC)), if conditioning regimen contained total body irradiation (TBI) or high-dose busulfan, type of immunosuppression, if immunosuppression contained anti-thymocyte globulin (ATG) or post-transplant cyclophosphamide (PTCy) and occurrence of acute GvHD.

If patients had been diagnosed with SOS, further data was collected such as lab parameters from start conditioning until resolution of symptoms or death (transaminases, creatinine, bilirubin), clinical course and outcome (clinical symptoms, time of onset, time to diagnosis, biopsy or autopsy confirmed SOS, treatment, occurrence of MOF). In all patients diagnosed with SOS with the modified Seattle criteria, additionally the EBMT diagnosis criteria and severity criteria for SOS were retrospectively applied [2].

With access to data from an accounting program, a sub-analysis from 01.01.2016 to 22.09.2020 could be performed. All patients who received DF (for treatment or prophylaxis) in this period could be extracted and the EBMT diagnosis criteria for SOS were applied retrospectively.

The primary endpoint of the study was the incidence of SOS in the period examined, calculated as cumulative incidence 6 months after HSCT. Secondary, uni- and multivariate analysis were performed to detect potential risk factors for SOS development. The overall survival (OS), progression-free survival (PFS), incidence of non-relapse-mortality (NRM) and relapse one year after transplantation were calculated for patients with and without SOS. The day+100 survival was calculated for SOS patients. In the sub-analysis for 2016–2020, the occurrence of SOS was analysed depending on the provision of DF. The study was approved by the Ethics Committee of Northwestern and Central Switzerland (EKNZ study number: 2020-02784).

### Conditioning regimen and GvHD prophylaxis

MAC protocols consisted of cyclophosphamide combined with busulfan, cyclophosphamide and TBI ≥8 Gy, cytarabine, carmustine, etoposide, melphalan, and fludarabine (BEAM- fludarabine) and further protocols [28]. RIC protocols included fludarabine with low-dose TBI (<6 Gy), fludarabine combined with busulfan or melphalan, and other protocols. Reasons for RIC were relevant comorbidities or older age [28]. Conditioning regimen containing high dose busulfan was defined by administration of >8 mg busulfan/kg [29].

Within MAC conditioning regimens, GvHD prophylaxis consisted of cyclosporine A (CsA) and methotrexate (MTX) or other protocols. In patients with RIC containing fludarabine and busulfan the GvHD prophylaxis included CsA and MTX, if RIC was based on fludarabine and low-dose TBI it contained CsA and mycophenolate mofetil (MMF) [28]. In case of haploidentical donor the GvHD prophylaxis consisted of PTCy, CsA, and MMF. Generally, ATG was administered if the donor was unrelated or matched but donor or recipient were ≥40 years old [30]. Acute GvHD was diagnosed clinically and confirmed histologically if possible, and was graded according to the modified Glucksberg criteria [31]. In case of clinically relevant acute GvHD grade ≥2, patients were treated with corticosteroids i.v. (methylprednisolone, 2 mg/kg/d) [32].

### Transfusion support and infection prophylaxis

Red blood cell transfusion was given to patients with hemoglobin concentrations <70 g/l, platelet transfusion if the level was <10 G/l, or <20 G/L, in case of bleeding, mucositis, or fever. Generally, patients received antiviral prophylaxis with valaciclovir (500 mg/day orally) until day+30, prophylaxis against Pneumocystis jirovecii and Toxoplasmosis with trimethoprim/sulfamethoxazole (160/800 mg, three times weekly, orally) at least for 6 months after HSCT, and fluconazole (400 mg once weekly orally) prophylactically against yeast infections until day + 30. Most patients did not receive mold-active prophylaxis but were treated empirically or pre-emptively, following a diagnostic-driven approach, based on chest CT scans and serum galactomannan that were performed regularly [33].

### SOS diagnosis, prophylaxis and treatment

SOS diagnosis was made by the treating physician after HSCT by clinical criteria, using modified Seattle [2] and from 2016 both were applied; modified Seattle criteria and EBTM-criteria [4]. Only a few cases were confirmed by liver biopsy due to the high risk of the invasive procedure, one patient was diagnosed post mortem by autopsy.

Date of diagnosis of SOS was defined by the day when the treating clinician made the diagnosis. Date of SOS onset was defined by the first day with bilirubin level ≥2 mg/dl (34 µ/ml), or first documentation of ascites or weight gain >5% without another explanation, retrospectively.

MOF was defined by dysfunction of two or more organs: renal failure (creatinine >2x baseline or GFR < 50% of baseline), pulmonary failure (saturation of peripheral oxygen (Spo2) <90% room air or/and need for positive pressure or ventilation dependence not attributable to any other cause) or CNS failure (confusion, lethargy or delirium not attributable to any other cause) [4].

Oral UDCA (3×250 mg daily) and continuous infusion of low-dose heparin (5000 IE/day) was used as SOS prophylaxis and was usually started simultaneously as the conditioning regimen and stopped after engraftment and when liver values were near the normal range. Low-dose Heparin was administered without monitoring, and only stopped in case of major bleeding or treatment initiation with DF. In selected cases with increased risk for SOS (e.g. patients with impaired liver function or second allo-HSCT), DF was administered as SOS prophylaxis according to the treating physician’s decision. When DF was administered for prophylaxis or treatment, dose of 6.25 mg/kg every 6 h was given intravenously., and low-dose heparin was stopped. DF was administered until resolution of symptoms or until engraftment, if given prophylactically.

### Statistical analysis

Continuous variables are reported as median (minimum to maximum) and categorical variables are presented as absolute values and percentages. We assessed univariable differences between patients with and without SOS with regard to patient, disease or transplant characteristics, testing statistical significance by Chi-squared or Fisher’s exact test for categorical variables and by the Mann–Whitney U test for continuous variables. A multivariable analysis was then performed using logistic regression analysis. Variables were included in the model using a backward selection by likelihood ratio test. Results were expressed as odds ratio (OR) with 95% confidence interval (CI). OS and PFS were calculated using the Kaplan-Meier method. Comparisons of OS and PFS were performed using the log-rank test. OS was calculated from allo-HSCT until death from any cause or last follow up, with censoring of survivors. PFS was calculated from allo-HSCT to relapse, death or last follow up, with censoring of relapse free survivors. NRM was defined as death without prior relapse. Cumulative incidence curves for relapse and NRM were constructed considering NRM or relapse as a competing event, respectively, using the Fine and Gray method to test for difference. All *p* values are two-sided and *p* < 0.05 was considered statistically significant.

Statistical analysis was performed using the SPSS (Statistics v25 IBM, Chicago, IL, USA) and Stata (SE v16 StataCorp LLC, College Station, TX, USA) software.

## Results

### Patient and disease characteristics

Between 2006 and 2020, 1,163 allo-HSCTs were performed at the University Hospital of Basel. In total, *n* = 1,016 patients were included in the final analysis (Fig. 1). The median observation time of the entire patient cohort was 563 days (0–5217). The median age was 54 years, ranging from 18–76 (Table 1). Compared to the period between 2006 and 2013, a slightly greater proportion of the patients received the allo-HSCT between 2013 and 2020 (56.9%). More men than women received allo-HSCT (61.8%), and the most frequent underlying disease was acute myeloid leukemia (AML) (38.6%). Most patients received their first allo-HSCT (70.8%) and had a KPS of 80% or more (or ECOG from 0 to 1 respectively) (89.3%). The majority underwent MAC (69.2%) and the most used immunosuppression was CsA with MTX (62.9%). ATG was administered to 40.2% of the total cohort and in 33.1% acute GvHD occurred with grade 2 or more. All patient and transplant-related characteristics are described in Table 1.

**Table 1:** Baseline Patient Characteristics, Univariable Analysis of Association with SOS Development.

Variable	No SOS (*n* = 993)	SOS (*n* = 23)	All patients (*n* = 1,016)	*p* value
Age (years), median (min - max)	54 (18–76)	49 (29–70)	54 (18–76)	0.161
	*n* (%)	*n* (%)	*n* (%)	
Age categories (years)				0.574
<40	217 (21.9)	6 (26.1)	223 (21.9)	
40–60	458 (46.1)	12 (52.2)	470 (46.3)	
>60	318 (32.0)	5 (21.7)	323 (31.8)	
Year of allo-HSCT				0.525
2006–2013	430 (43.3)	8 (34.8)	438 (43.1)	
2013–2020	563 (56.7)	15 (65.2)	578 (56.9)	
Sex				1.000
Male	614 (61.8)	14 (60.9)	628 (61.8)	
Female	379 (38.2)	9 (39.1)	388 (38.2)	
Diagnosis				0.849
AML	384 (38.7)	8 (34.8)	392 (38.6)	
ALL	123 (12.4)	3 (13.0)	126 (12.4)	
Lymphoma or Plasma Cell disorder	200 (20.1)	5 (21.7)	205 (20.2)	
MDS& MPN	245 (24.7)	7 (30.4)	252 (24.8)	
Bone marrow failure & others	41 (4.1)	0 (0)	41 (4.0)	
Disease status at allo-HSCT				0.0001
CR 1 or 2	574 (57.8)	4 (17.4)	578 (56.9)	
Advanced disease	419 (42.2)	19 (82.6)	438 (43.1)	
Previous HSCT (allogenic or autologous)				0.009
No	709 (71.4)	10 (43.5)	719 (70.8)	
Yes	284 (28.6)	13 (56.5)	297 (29.2)	
Type of previous HSCT				0.017
None	709 (71.4)	10 (43.5)	719 (70.8)	
Allogenic	87 (8.8)	3 (13.0)	90 (8.9)	
Autologous	187 (18.8)	10 (43.5)	197 (19.4)	
Allogenic and Autologous	10 (1.0)	0 (0)	10 (1.0)	
Performance status				0.511
Karnofsky ≥80% or ECOG 0-1	888 (89.4)	19 (82.6)	907 (89.3)	
Karnofsky <80% or ECOG 2-3	101 (10.2)	4 (17.4)	105 (10.3)	
Missing	4 (0.4)	0 (0)	4 (0.4)	
Donor				0.654
Matched relative	358 (36.0)	7 (30.5)	365 (35.9)	
Unrelated	554 (55.8)	13 (56.5)	567 (55.8)	
Haploidentical	81 (8.2)	3 (13.0)	84 (8.3)	
Donor sex				0.130
Male	602 (60.6)	10 (43.5)	612 (60.2)	
Female	391 (39.4)	13 (56.5)	404 (39.8)	
Conditioning regimen				0.493
Myeloablative	685 (69.0)	18 (78.3)	703 (69.2)	
Reduced intensity	308 (31.0)	5 (21.7)	313 (30.8)	
Conditioning with high dose Busulfan (>8 mg/kg)				1.000
No	685 (69.0)	16 (69.6)	701 (69.0)	
Yes	308 (31.0)	7 (30.4)	315 (31.0)	
TBI				0.481
No	625 (62.9)	16 (69.6)	641 (63.1)	
≥8 Gy	155 (15.6)	5 (21.7)	160 (15.7)	
<8 Gy	201 (20.2)	2 (8.7)	203 (20.0)	
TBI dose unknown	12 (1.2)	0 (0)	12 (1.2)	
GvHD Prophylaxis				0.065
CsA	49 (4.9)	4 (17.4)	53 (5.2)	
CsA + MTX	627 (63.1)	12 (52.2)	639 (62.9)	
CsA + MMF	242 (24.4)	5 (21.7)	247 (24.3)	
Other	75 (7.6)	2 (8.7)	77 (7.6)	
ATG used				0.395
No	592 (59.6)	16 (69.6)	608 (59.8)	
Yes	401 (40.4)	7 (30.4)	408 (40.2)	
PTCy used				0.059
No	930 (93.7)	19 (82.6)	949 (93.4)	
Yes	63 (6.3)	4 (17.4)	67 (6.6)	
aGvHD ≥ Grade 2				0.826
No	665 (67.0)	15 (65.2)	680 (66.9)	
Yes	328 (33.0)	8 (34.8)	336 (33.1)	

*SOS* sinusoidal obstruction syndrome, *min* minimum, *max* maximum, *allo-HSCT* allogenic hematopoietic stem-cell transplantation, *AML* acute myeloid leukaemia, *ALL* acute lymphoid leukemia, *MDS* myelodysplastic syndrome, *MPN* myeloproliferative neoplasm, *CR* complete remission, *ECOG* Eastern Cooperative Oncology Group, *TBI* total body irradiation, *GvHD* graft versus host disease, *CsA* cyclosporin A, *MTX* methotrexate, *MMF* mycophenolate mofetil, *ATG* Anti-thymocyte globulin, *PTCy* post-transplant cyclophosphamid, *aGvHD* acute graft versus host disease.

### Incidence of SOS and clinical course

Out of 1,016 patients, 23 developed SOS, with a cumulative incidence of 2.3% (95% CI 1.3–3.3) six months after HSCT. The median time to diagnosis was 14 days (3–151) and the median time to onset nine days (1–150). Approximately one quarter of the patients (*n* = 6, 26.1%) had late-onset SOS. Almost all patients had a weight gain >5% (*n* = 22, 95.7%), in most of them, ascites was detected (*n* = 19, 82.6%), and most suffered from liver pain (*n* = 18, 78.3%). Nineteen patients (82.6%) were treated with DF for a median of eight days (2–32). Thereof, two patients (8.7%) received DF already before, as a prophylactic treatment.

Twenty-two patients (95.7%) fulfilled the EBMT-criteria retrospectively, only one patient did not, due to lower bilirubin levels than 2 mg/dl. However, in this case the modified Seattle criteria were met. In nine patients (39.1%) the diagnosis was confirmed by biopsy or autopsy. The EBMT severity grading very severe was clearly the most frequent (*n* = 17, 73.9%) compared to severe (*n* = 2, 8.7%), moderate (*n* = 3, 13.0%) and mild (*n* = 1, 4.3%). In 69.6% (*n* = 16) of the cases MOF occurred. The overall survival on day + 100 of the SOS patients was 39.1% (95% CI 18.7–59.5). A more detailed presentation of the clinical course of the patients who developed SOS is displayed in Supplementary figs. 1A (17 patients with classical SOS), and 1B (six patients with late onset SOS).

### Subanalysis Defibrotide Administration from 2016 to 2020

From 2016 until 2020, 430 patients undergoing allo-HSCT could be included in the sub-analysis on DF provision. Among them, 20 patients (4.7%) received DF as a treatment for suspected SOS. Of these, 12 patients (60%) were finally diagnosed with SOS (fulfilling the EBMT-criteria retrospectively), corresponding to a cumulative incidence of 2.8% (95% CI 1.2–4.4) six months after HSCT. DF as a SOS prophylaxis had been administered to 17 patients (4.0%), thereof two patients developed SOS (11.8% of the prophylactically treated patients). Of notice: in total, only 35 patients received DF, the two patients developing SOS under prophylaxis were counted in both groups (treatment and prophylaxis).

### Risk factors for SOS

Both in univariable analysis (Table 1) and confirmed in multivariable analysis (Table 2), advanced disease (*p* = 0.003) and previous HSCT (allogenic or autologous) (*p* = 0.025) were significantly associated with the development of SOS. An association was also found between the use of PTCy and the development of SOS, although it was not statistically significant (*p* = 0.055).

**Table 2:** Multivariable Analysis of Risk Factors for SOS (*n* = 1,016).

Variable	OR (95% CI)	*p* value
Previous HSCT (allogenic or autologous)		
Yes vs. No	2.68 (1.13–6.34)	0.025
Disease status at allo-HSCT		
Advanced disease vs. CR1 or 2	5.26 (1.77–15.86)	0.003
PTCy used		
Yes vs. No	3.07 (0.98–9.61)	0.055

*SOS* sinusoidal obstruction syndrome, *OR* odds ratio, *CI* confidence interval, *allo-HSCT* allogenic hematopoietic stem-cell transplantation, *PTCy* post-transplant cyclophosphamide.

### Survival, PFS, NRM and relapse

**Fig. 2:**
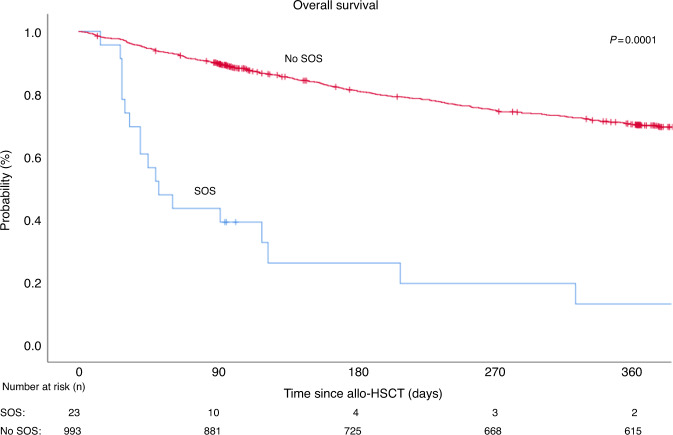
Survival after allogeneic transplant. Overall Survival. Patients with SOS vs. no SOS, 2006–2020.

**Fig. 3:**
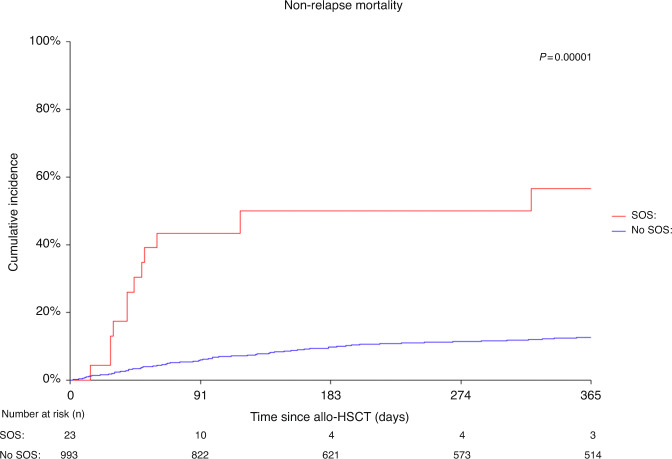
Non-relapse Mortality. Patients with SOS vs. no SOS, 2006–2020 (*p* = 0.00001).

As shown in Table 3 and displayed in the Figs. 2 and 3, the outcomes were less favorable in the SOS group: The 1-year OS was significantly lower in the SOS group with 13% (95% CI −3.6 to 30) compared to the patients without SOS with 70% (95% CI: 67–73) (*p* = 0.0001) (Fig. 2). PFS was also lower in the SOS group with 13% (95% CI −3.6 to 30) versus 60% (95% CI 57–63) (*p* = 0.0001) (Supplementary Fig. 2). The cumulative incidence of the one-year NRM was significantly higher in the SOS group with 57% (95% CI 38–83), in comparison to the non-SOS group with 13% (95% CI 11–15)) (*p* = 0.000001) (Fig. 3), whereas no difference was seen in the cumulative incidence of one-year relapse (Supplementary Fig. 3).

**Table 3:** Allo-HSCT outcome at one year in patients with SOS vs. patients without SOS.

Variable	SOS (*n* = 23)	No SOS (*n* = 993)	*p* value
	% (95% CI)	% (95% CI)	
1-year OS	13 (−3.6 to 30)	70 (67–73)	0.0001
1-year PFS	13 (−3.6 to 30)	60 (57–63)	0.0001
1-year NRM CIF	57 (38–83)	13 (11–15)	0.000001
1-year relapse CIF	30 (15–60)	27 (24–30)	0.982

*Allo-HSCT* allogenic hematopoietic stem-cell transplantation, *SOS* sinusoidal obstruction syndrome, *CI* confidence interval, *OS* overall survival, *PFS* progression-free survival, *NRM* non-relapse mortality, *CIF* cumulative incidence function.

## Discussion

Firstly described forty years ago, as a complication after allo-HSCT, SOS remains a complication with high mortality until today [1, 34]. The purpose of this study was to investigate the incidence of SOS after allo-HSCT in patients receiving low-dose heparin and UDCA as SOS prophylaxis. We found a low incidence of SOS with 2.3%, but a high mortality, with only 39% survival at day + 100, although a high proportion of SOS patients were treated with DF (83%). A comparable overall incidence of SOS of 3.4% in adults after allo-HSCT was reported in a transplantation center in Korea following a similar SOS prophylaxis strategy (prophylactic UDCA and intravenous heparin, or prostaglandin E1 in selected cases) [9]. These results suggest that prophylactic UDCA and low-dose heparin might successfully prevent SOS cases. Given to the current state of research with inconclusive results concerning low-dose anticoagulation in SOS prophylaxis and current recommendations are mainly based on expert opinion [22, 35], new RCTs are warranted to assess the benefit of prophylactic anticoagulation for SOS. The claim for RTCs is thus substantiated given that there are now two transplantation centers reporting comparable results for study populations (*n* = 2,572 [9] and *n* = 1,016 (the present work)).

In recent studies, the SOS incidence after allo-HSCT varied from 7.5% (patients 15 years and older) [36], to 5.2% (at day + 100 after allo-HSCT in adults) [37], to 2.9% (children and adults) [38]. Due to the heterogeneity of the composition of the study populations (e.g. adults or children only versus both, MAC or RIC versus both), comparisons of the incidence across studies are difficult. Children for instance seem to have a higher incidence for SOS [2], and MAC is a well-described risk factor [1, 5]. Nevertheless, there seems to be a trend towards a reduction of the SOS frequency over time [39], which is consistent with the finding of the low SOS incidence in our study. The reduction could be related to the increasing use of RIC [37], a decreasing use of double alkylator busulfan regimens [6] and the more often used pharmacokinetic-guided individualized dosing of busulfan [39]. The number of allo-HSCT globally performed is however steadily increasing [40], as well as the transplantations performed in more heavily pretreated patients [1], implicating that SOS will remain an important complication after HSCT.

A possible explanation for the low survival at day+100 of the SOS patients in our study is the high proportion of severe and very severe SOS cases (83%). Our finding is similar to the survival rates at day + 100 of patients with severe or very severe SOS described in literature [11, 12, 41]. Also, the significantly lower OS, PFS and higher NRM of the SOS patients reflect the impact of SOS on mortality after HSCT.

In the current study, 26% were late onset SOS cases, which is consistent with existing literature [37, 38], as well as the median time to diagnosis (day + 14) [12, 36, 39]. One patient was diagnosed markedly later than the other SOS cases (day+151), shortly after administration of gemtuzumab ozogamicin. This agent has been multiply described as risk factor for SOS [1, 2], but lately Ho et al. published opposite results [42].

As presented in Supplementary fig. 1A and B, the timeline of the clinical course of SOS cases is heterogeneous, illustrating the difficulty of diagnosis. In addition, to differentiate SOS from other complications, as drug induced liver toxicity, disseminated intravascular coagulation, transplant-associated thrombotic microangiopathy (TA-TMA) or hepatic acute GvHD, poses a challenge for early diagnosis. Despite the variability of the clinical course, nearly all cases met the EBMT diagnosis criteria (96%), which underlines their applicability.

The risk factors significantly associated with SOS development in our multivariable analysis are already well described in literature (advanced disease [15, 43] and previous stem cell transplantation [1, 43]). In our study, PTCy correlated also with an increased, but not significant, risk for SOS. Now, there is no literature available about post-HSCT GvHD prophylaxis with PTCy as a risk factor for SOS. Further research is needed to evaluate this association.

A systematic review analyzing studies using DF as SOS prophylaxis reported a mean SOS incidence of 5% (only abstract available) [44]. A small retrospective study reported a SOS incidence of 6.3% (*n* = 4/63) in patients considered at high risk for SOS receiving prophylactic DF [45]. In our study (sub-analysis 2016–2020) two out of seventeen patients receiving DF prophylactically were diagnosed with SOS (11.8%). Due to this small number of patients, no conclusive statement about DF as SOS prophylaxis can be made. Still, we can report the observation that SOS also occurs after application of prophylactic DF, and also at an actually higher SOS rate in the group receiving DF prophylactically (*n* = 2/17, 11.8%), compared to the patients with standard SOS prophylaxis (heparin, UDCA) (*n* = 10/413, 2.4%). It is noteworthy, that the patients receiving DF prophylactically were considered as high risk patients for SOS. Given the lack of data for DF as SOS prophylaxis in adults, more research is needed, especially when taking into account the high costs of the agent. UDCA and low-dose heparin are, in contrary, a low-price SOS prophylaxis.

Our study has several draw backs. Most importantly, a control group is lacking. Due to the practically non-existent side effects of UDCA and low-dose heparin, these were administered to nearly all patients, assuming that the benefits are outweighing potential side effects. Another main limitation of the study is the observational and retrospective setting, which may be related to the markedly high proportion of severe and very severe SOS cases. We cannot exclude a potential underdiagnosis of mild SOS cases. Furthermore, given the low number of identified SOS cases, the power for assessing risk factors was limited.

In conclusion low-dose heparin and UDCA might be a promising low-cost prophylactic approach to prevent SOS, although the mortality of SOS cases remains very high, even when they are treated with DF. Further research is warranted on SOS prophylaxis and treatment, especially prospective and randomized trials.

## Supplementary information

Supplementary material

## Data Availability

The datasets generated during and/or analysed during the current study are available from the corresponding author on reasonable request.
