# Supplementary material for: Low Incidence of hepatic sinusoidal obstruction syndrome/veno-occlusive disease in adults undergoing allogenic stem cell transplantation with prophylactic ursodiol and low-dose heparin
Source: Bone Marrow Transplant. 2022 Jan 4;57(3):391–8. doi: 10.1038/s41409-021-01546-w (PMC8907064; doi:10.1038/s41409-021-01546-w)

**Supplementary Figure 1A:** Clinical course of classical SOS cases (n=17)

**Supplementary Figure 1B:** Clinical course of late onset SOS cases (n=6)

**Supplementary Figure 2:** Progression-free Survival SOS vs. no SOS, 2006 - 2020


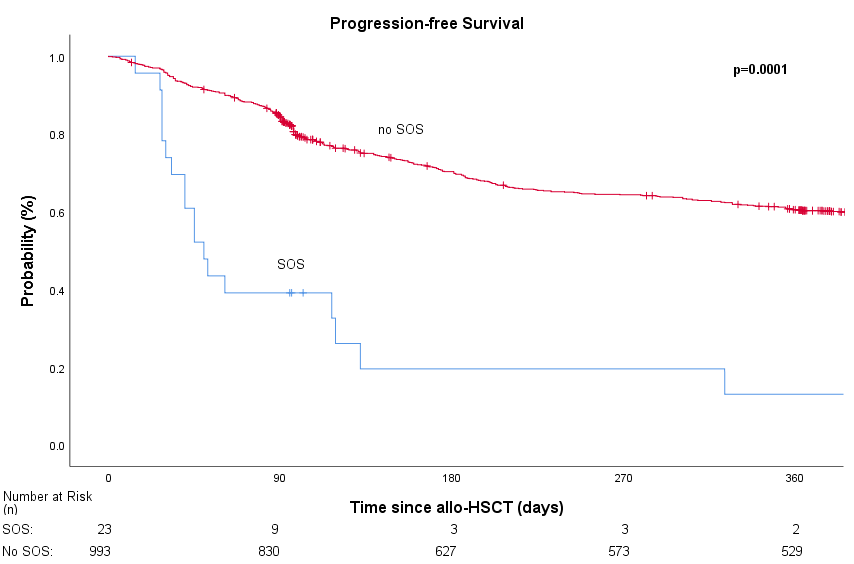


**Supplementary Figure 3:** Relapse SOS vs. no SOS, 2006 - 2020 (p=0.982)


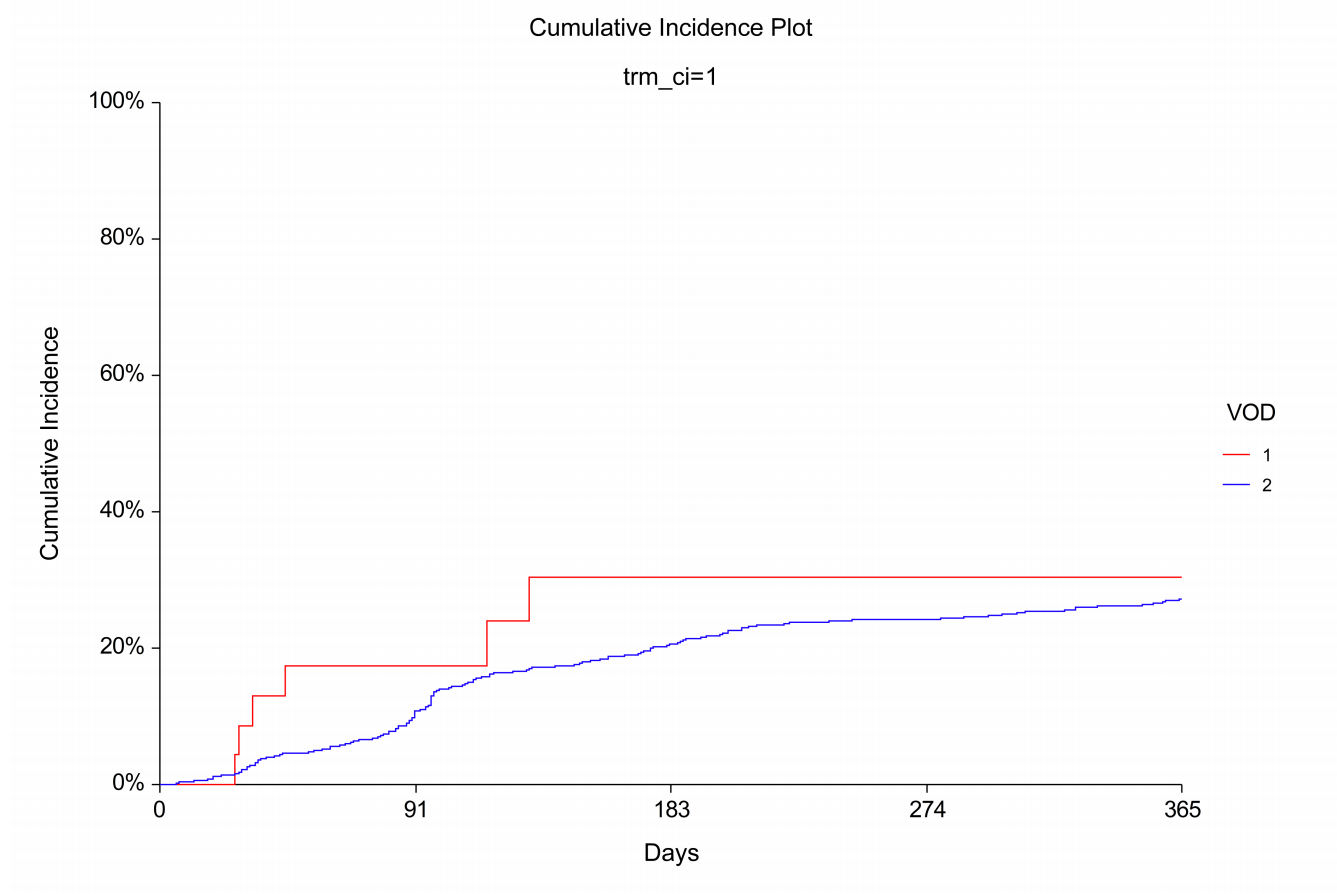

Supplement: Supplementary file 1 — Supplementary material [file 41409_2021_1546_MOESM1_ESM.docx]
